# Gi‐DREADD activation decreases Epithelial Na^+^ channel activity in renal principal cells

**DOI:** 10.14814/phy2.70433

**Published:** 2025-06-17

**Authors:** Tarek Mohamed Abd El‐Aziz, Elena Mironova, James D. Stockand, Lucia A. Seale, Antonio G. Soares

**Affiliations:** ^1^ Zoology Department, Faculty of Science Minia University Minya Egypt; ^2^ Greehey Children's Cancer Research Institute University of Texas Health Science Center San Antonio Texas USA; ^3^ Cellular and Integrative Physiology Department, The University of Texas Health Science Center at San Antonio San Antonio Texas USA; ^4^ Pacific Biosciences Research Center, University of Hawaiʻi at Mānoa Honolulu Hawaii USA

**Keywords:** epithelial sodium channel, Gi‐DREADD, sodium excretion

## Abstract

The activity of the Epithelial Na^+^ Channel (ENaC) in renal principal cells (PC) fine‐tunes sodium excretion and consequently affects blood pressure. G‐coupled receptors play an important role in regulating ENaC activity. We previously explored the role of Gq and Gs in regulating ENaC activity by using the designer receptors exclusively activated by designer drugs (DREADD) technology. We demonstrated that pharmacogenetic activation of Gq (Gq‐DREADD) exclusively in principal cells by Clozapine‐N‐oxide (CNO) reduced ENaC activity in renal tubules, promoting natriuresis that lowered elevated blood pressure in the DOCA‐salt model of hypertension. In addition, by investigating the Gs‐adenylyl cyclase‐cAMP signal transduction pathway, we exhibited that treatment of PC‐specific Gs‐DREADD mice with CNO rapidly and significantly decreased urinary Na^+^ excretion. In this study, we investigate the role of Gi‐DREADD in regulating ENaC activity. Our results showed that Gi‐DREADD, expressed exclusively in renal principal cells, activated by CNO reduced ENaC activity and significantly increased urinary Na^+^ excretion compared to CNO‐treated littermates. These findings provide for the first time that target activation of Gi signaling exclusively in PCs is sufficient to decrease ENaC activity and increase dependent urinary Na^+^ excretion in live animals.

## Graphical abstract

Vasopressin, acting via Gs‐signaling pathway increases the activity of the epithelial Na+ channel (ENaC). Activation of the Gi‐DREADD receptor expressed exclusively in renal principal cells of the collecting duct is sufficient to reduce ENaC activity, therefore, increasing Na+ excretion.

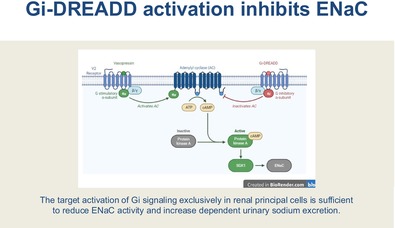

## 1 INTRODUCTION

The Epithelial Na^+^ Channel (ENaC) is a crucial ion channel, composed of three homologous subunits: α, β, and γ. It plays a significant role in sodium reabsorption within the renal system, particularly in the distal nephron, which includes the late distal convoluted tubule, connecting tubule, and collecting duct (Pearce et al., 2022). ENaC activity is important for maintaining sodium homeostasis, regulating extracellular fluid volume, and fine‐tuning blood pressure (Schild, 2010; Verouti et al., 2015). Hormones such as vasopressin potently stimulate ENaC activity through a G protein‐coupled receptor (GPCR) pathway (Kleyman & Eaton, 2020; Pearce et al., 2022). GPCR are a large and diverse group of membrane proteins that play a crucial role in cellular communication by transducing extracellular signals into intracellular responses. The Gα subunits (Gi, Gq, and Gs) can activate distinct signaling pathways influencing renal functions such as glomerular filtration, tubular reabsorp*t*ion, and the regulation of blood pressure (Park, 2015). This pathway activates adenylyl cyclase, elevating cyclic AMP (cAMP) levels, which subsequently promotes ENaC activity by facilitating its insertion into the apical membrane of principal cells in the aldosterone‐sensitive distal nephron (Castañeda‐Bueno & Ellison, 2022).

Previous work from our group sought to elucidate how GPCR could modulate ENaC activity. We demonstrated that activation of P2Y2 receptors, which coupled to Gq, stimulates phospholipase C (PLC), leading to intracellular calcium mobilization and subsequent ENaC inhibition. The designer receptors exclusively activated by designer drugs (DREADD) technology proves to be an outstanding tool to dissect GPCR signaling as one can investigate Gq, Gs, and Gi without interference (Zhu & Roth, 2014). By utilizing a Gq‐DREADD mouse model, we demonstrated that Gq signaling is sufficient to promote sodium excretion by inhibiting ENaC activity (Mironova et al., 2019; Soares et al., 2023). Such observations are important as P2Y2 receptor knockout mouse models increased ENaC activity, which contributed to salt‐sensitive hypertension (Pochynyuk et al., 2010; Vallon et al., 2012). These observations emphasized the crucial role of Gq‐coupled receptors in regulating ENaC activity, sodium balance, and blood pressure.

In addition, we demonstrated that the Gs‐adenylyl cyclase‐cAMP signaling pathway is integral to the regulation of ENaC. By employing the Gs‐DREADD mouse model, a knock‐in model expressing Gs‐DREADD with a cAMP response element‐luciferase reporter transgene for non‐invasive bioluminescence monitoring of cAMP signaling, we demonstrated that exclusive activation of Gs signaling in renal principal cells is sufficient to rapidly increase ENaC activity to reduce Na^+^ excretion similar to that observed by vasopressin (Soares et al., 2021), providing insights into how this signaling pathway modulates ENaC activity and, consequently, sodium balance.

Building upon these findings, we questioned the role of Gi in regulating ENaC activity and renal sodium handling. It is well established that Gi subunits can inhibit adenylate cyclase, leading to reduced levels of cyclic AMP, which affect renal tubular function and fluid balance (Ren et al., 2013). In addition, Gi‐coupled receptors are involved in the prostaglandin E2 signaling (Mutsaers & Nørregaard, 2022). However, it is unknown whether Gi can directly modulate ENaC activity. To investigate the role of Gi in regulating ENaC, we generated a knock‐in mouse expressing the Gi‐DREADD exclusively in renal principal cells (PC). Our results demonstrated that activation of Gi‐DREADD in renal principal cells by Clozapine‐N‐oxide (CNO) was sufficient to increase sodium excretion and to reduce ENaC activity. These findings demonstrate for the first time that target activation of Gi signaling exclusively in PCs is sufficient to decrease ENaC activity and increase dependent urinary Na^+^ excretion in vivo.

## 2 METHODS

### 2.1 Animals

All animal use and welfare complied with the National Institutes of Health *Guide for the Care and Use of Laboratory Animals*. Protocols were reviewed and approved by the Institutional Animal Care and Use Committee of the University of Texas Health Science Center at San Antonio. Mice were housed and cared for in the Laboratory Animal Resources Facility at the University of Texas Health Science Center at San Antonio, which is fully accredited by the Association for Assessment and Accreditation of Laboratory Animal Care and licensed by the United States Department of Agriculture. Results involving animal studies were in compliance with ARRIVE (Animal Research: Reporting of in vivo Experiments) guidelines. Healthy young adult (2–3 months, 19.82 ± 0.16 g body weight) male and female mice were used in this study. Experimental mice had PC‐specific expression of Gi‐DREADD and control mice were either wild type (WT), Aqp2‐cre negative, or Gi‐DREADD‐negative littermates. All mice were housed under standard 12‐h light/dark cycles at room temperature and had ad libitum access to water and standard chow (Teklad LM‐485 Mouse/Rat Sterilizable Diet, Cat. No. T.7012.15; Envigo BioProducts, Indianapolis, IN) throughout the acclimation and experimental periods.

### 2.2 Creation of the principal cell‐specific Gi‐DREADD mouse

**FIGURE 1:**
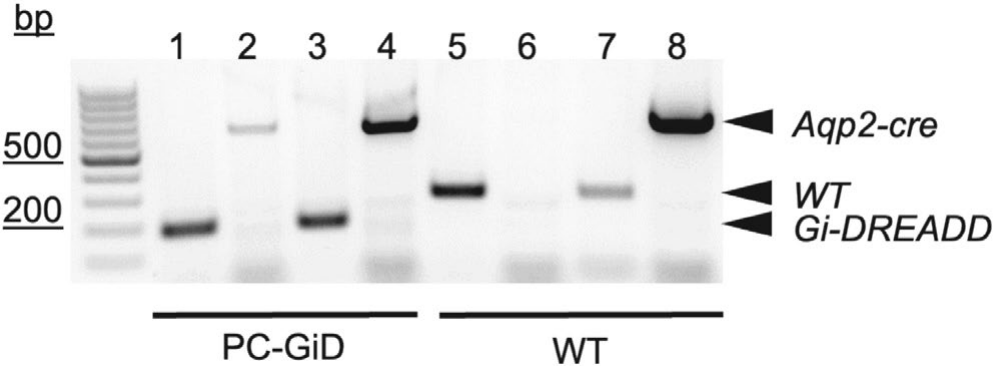
Genotyping of Principal cell‐specific Gi‐DREADD mice. Representative gel containing PCR products from genotyping for PC‐GiD mice lanes 1 and 3, and wild type (WT) littermate controls (5 and 7). Products for the Aqp2‐Cre transgene are shown in lanes 2, 4, and 8. Lane 6 indicates a WT control lacking the Aqp2‐Cre transgene. To increase visual clarity without changing content, contrast and brightness were adjusted, and the gray scale of this digital image was inverted. bp, base pairs.

Target expression of Gi‐DREADD in renal principal cells (PC‐GiD) was achieved by crossing male B6N.129‐Gt(ROSA)26Sor^tm1(CAG‐CHRM4*,‐mCitrine)Ute^/J with female B6.Cg‐Tg(Aqp2‐cre)1Dek/J mice, and C57Bl/6 was used as wild type controls. No noticeable difference in behavior, body weight, pathology, or any other gross attribute was observed between Gi‐DREADD:Aqp2‐cre and littermate controls. For genotyping reactions, the Gi‐DREADD transgene presence was confirmed with the forward 5′‐CGAAGTTATTAGGTCCCTCGAC‐3′ and reverse 5′‐TCATAGCGATTGTGGGATGA‐3′ PCR primers, producing an expected 200‐bp product (Figure 1). The WT allele, which lacked insertion of the Gi‐DREADD transgene, was identified with the forward 5′‐AAGGGAGCTGCAGTGGAGTA‐3′ and reverse 5′‐CCGAAAATCTGTGGGAAGTC‐3′ PCR primers producing an expected 297‐bp product. The Aqp2‐cre transgene was identified with the forward 5′‐CTCTGCAGGAACTGGTGCTGG‐3′ and reverse 5′‐GCGAACATCTTCAGGTTCTGCGG‐3′ PCR primers, producing an expected 673‐bp product.

### 2.3 Urine excretion assessment

Metabolic cages (Techniplast, Buguggiate, Italy) quantified excretion over a 24 h period following previously published protocols with minor modifications (Soares et al., 2021). In brief, age and weight matched PC‐ GiD and littermate control mice were single‐housed in metabolic cages and allowed to acclimate for 2 days. On the third day, 24 h urines were collected before Clozapine‐N‐oxide (CNO) injection. After 24 h, urine was collected again on the fourth day following injection with CNO (0.1 mg/kg; s.c.). Collection surfaces in contact with urine were collected under light mineral oil to increase the precision of the measurements by reducing the resistance to flow to the final collecting reservoir and to minimize loss due to evaporation. Urinary Na^+^ (U_Na_V) concentration was quantified with a flame photometer (Jenway, Staffordshire, United Kingdom). Urinary [Na^+^] was multiplied by 24 h urine volume (V) to obtain excretion.

### 2.4 Single‐channel patch‐clamp electrophysiology

Split‐open tubules amenable to patch‐clamp analysis were prepared as previously described (Mironova et al., 2019). In brief, freshly isolated mouse kidneys were sectioned transversely. Segments of the CNT and CCD were manually microdissected with watchmaker forceps under a stereomicroscope and adhered to a glass chip coated with 0.01% poly‐lysine. These chips were then transferred to an inverted microscope, where tubules were split open with sharpened pipettes. Patch‐clamp electrophysiology was performed using an Axopatch 200B (Axon Instruments) patch‐clamp amplifier interfaced via a Digidata 1322A (Axon Instruments) to a PC running pClamp 9.2 software (Axon Instruments), and currents were low‐pass‐filtered at 100 Hz with an eight‐pole Bessel filter (Warner Instruments). Gap‐free single‐channel patch‐clamp experiments were run in the cell‐attached configuration at a holding potential of −60 mV; unitary current amplitude was approximately −1.8 pA and performed on the luminal plasma membranes of PCs in these split‐opened tubules following standard protocols (Mironova et al., 2019). Split‐open tubules were used within 1–2 h of isolation. The bath solution contained 150 mM NaCl, 5 mM KCl, 1 mM CaCl_2_, 2 mM MgCl_2_, 5 mM glucose, and 10 mM HEPES (pH 7.4), while the pipette solution contained 140 mM LiCl, 2 mM MgCl_2_, and 10 mM HEPES (pH 7.4). A current–voltage correlation was established for ENaC, which had a conductance of 4–5 pS with 140 mM NaCl in the pipette. The pipette resistance was approximately 7–10 mΩ. In these cell‐attached voltage‐clamp studies, current was low‐passed at 100 Hz. All single‐channel recordings in this study were conducted at a fixed holding potential of −60 mV. This potential is commonly used in ENaC electrophysiology because it provides a strong inward driving force for Na^+^, allowing for optimal resolution of inward single‐channel currents. While a full I–V curve was not generated, the use of a consistent holding potential enables accurate comparisons of a single‐channel current amplitude and open probability across experimental groups. Channel activity (NP_o_; open probability, P_o_, multiplied by channel number, N) was calculated as previously described (Mironova et al., 2019). Channel activity, defined as NPo, was calculated using the equation NPo = Σ(*t*_1_ + 2 *t*_2_ + … *it*_*i*_), where *t*_*i*_ is the fractional open time spent at each of the observed current levels. Open probability was estimated by normalizing NPo for the observed number of channels within a patch. The error that is associated with this estimation of the open probability increases as patches contain more channels and as open probability approaches either 0 or unity. ENaC activity was recorded in tubules pre‐treated with vehicle or CNO (2 μg/mL) for 30 min prior to patch‐clamp analysis.

### 2.5 Statistical analysis

Data were analyzed and plotted using GraphPad Prism 10 (GraphPad Software, Inc., San Diego, CA, United States). Values are reported as mean ± standard error of the mean (SEM). Data were compared using a two‐sample, two‐tailed or paired *t*‐test, or two‐way ANOVA as appropriate, and a *p* < 0.05 was considered significant.

### 2.6 Data availability statement

The authors declare that data and material are available upon reasonable request.

## 3 RESULTS

### 3.1 Targeted activation of Gi‐DREADD in principal cells increases Na^+^ excretion

**FIGURE 2:**
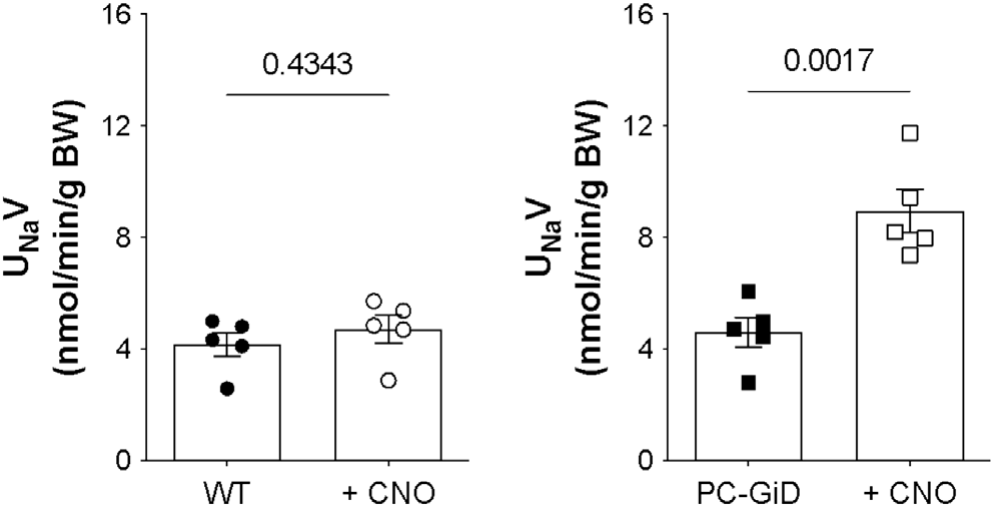
CNO treatment increases Na^+^ excretion in PC‐specific Gi‐DREADD mice. Summary graphs from paired experiments showing 24 h urinary Na^+^ excretion (U_Na_V) before and after treatment with CNO (0.1 mg/kg) in wild type littermate controls (A, *n* = 5/group) and PC‐specific GiD mice (B, *n* = 5/group).

After confirming the PC‐specific Gi‐DREADD genotype (Figure 1), we tested in vivo inhibition of ENaC. To understand the physiological consequences of ENaC inhibition by activation of the PC‐GiD in the living animal, we quantified CNO‐sensitive urinary Na^+^ excretion (U_Na_V) in control and PC‐specific GiD mice. Figure 2 shows summary results from paired experiments quantifying U_Na_V before and after treatment with CNO. Sodium excretion was not significantly affected by CNO in control mice (4.18 ± 0.42 vs. 4.72 ± 0.49 nmol/min/g BW; Figure 2a). As expected, CNO significantly increased U_Na_V in PCs‐specific GiD mice from 4.61 ± 0.53 to 8.95 ± 0.78 nmol/min/g BW (Figure 2b). Sodium excretion in PCs‐GiD mice before treatment with CNO was similar to that of untreated control mice. These data demonstrate that target activation of Gi‐DREADD in principal cells significantly increases urinary sodium excretion.

### 3.2 Targeted activation of Gi‐DREADD in principal cells decreases ENaC activity

**FIGURE 3:**
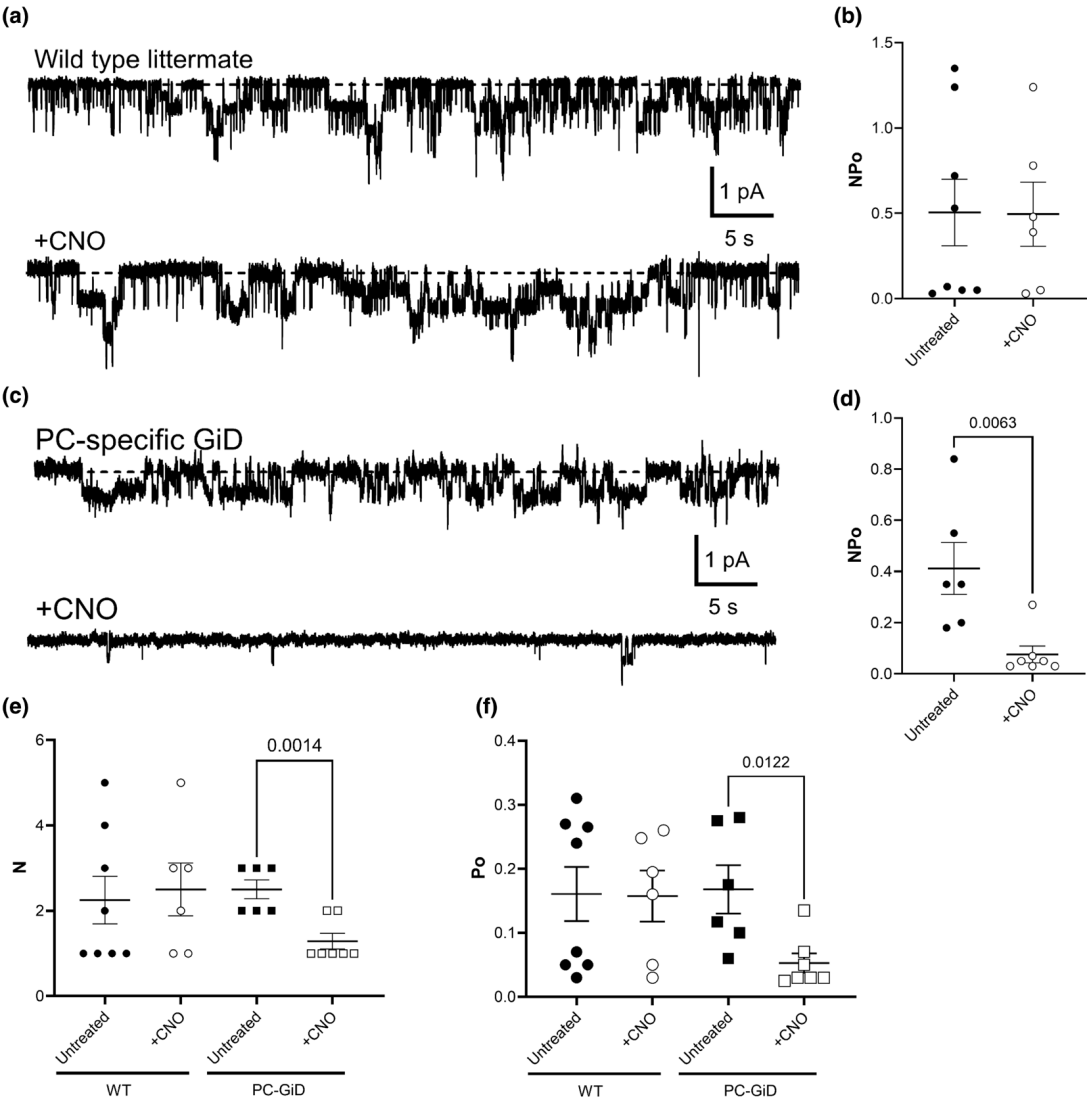
CNO decreases ENaC activity in principal cell‐specific Gi‐DREADD. All data represent cell‐attached patch‐clamp recordings of ENaC from the apical membranes of principal cells in freshly isolated tubules. Inward Na+ currents are depicted downwards with dashline indicating the closed state. For all graphs, +CNO indicates tubules where pre‐treated with CNO (2 μg/mL) for 30 min. Wild type data are represented by circles (untreated: Black and +CNO: White) and PC‐specific GiD represented by squares (untreated: Black and +CNO: White). (a & c) Show representative gap‐free ENaC current traces from wild type (a) and PC‐specific GiD. (b & d) Show summary graphs of ENaC activity (NPo) in principal cells from wild type controls (b) and PC‐specific GiD (d). (e & f) Show summary graphs of ENaC number N (e) and Po (f).

Next, we tested whether selective stimulation of Gi signaling in PC was sufficient to decrease ENaC activity. ENaC activity (NP_o_) in the apical plasma membranes of PCs in tubules isolated from littermate control and PC‐specific GiD mice was quantified in cell‐attached patches using patch‐clamp electrophysiology. Figure 3 shows representative current traces for ENaC in tubules isolated from control (Figure 3a) and PC‐specific GiD (Figure 3c) mice before (top) and after (bottom) treatment with CNO. Figure 3b shows that ENaC activity (NP_o_) in tubules from control mice was unaffected by treatment with CNO (0.50 ± 0.19 vs. 0.49 ± 0.18). In contrast, CNO significantly decreased ENaC activity in PCs in tubules isolated from PC‐specific GiD mice from 0.41 ± 0.10 to 0.07 ± 0.03 (Figure 3d). The activity of ENaC in tubules from PC‐specific GiD mice in the absence of CNO was not different than that in the littermate controls. Decreases in both ENaC number (N; 2.50 ± 0.22 vs. 1.29 ± 0.18; Figure 3e) and open probability (P_o_; 0.17 ± 0.04 vs. 0.05 ± 0.01; Figure 3f) drove activity decreases in PC‐specific GiD mice in response to CNO treatment. Our electrophysiological recordings specifically assessed ENaC activity in the collecting duct, and ENaC is known to be expressed exclusively in principal cells in this segment (Palmer, 1992). Therefore, the functional data inherently reflect activity from principal cells. These data demonstrate that selective stimulation of Gi signaling in principal cells is sufficient to decrease ENaC activity by reducing the number of active channels and their opening probability.

## 4 DISCUSSION

In this study, we have demonstrated for the first time that targeted stimulation of Gi signaling in renal PCs is sufficient to decrease ENaC activity and increase dependent urinary Na^+^ excretion in vivo. This is important as understanding the mechanisms associated with ENaC inhibition by Gi can aid in the development of targeted therapies aimed at modulating renal function and in the control of hypertension.

Gi proteins are a class of G proteins that inhibit adenylate cyclase, leading to reduced cAMP production. This cAMP decrease leads to reduced activity of protein kinase A (PKA), a key mediator of cAMP signaling. PKA typically phosphorylates various proteins involved in sodium transport, including ENaC and sodium‐potassium ATPase (Na^+^/K^+^‐ATPase). In addition, the interplay between Gi signaling and other pathways, such as those activated by vasopressin (Gs), illustrates the complexity of sodium transport regulation. Vasopressin increases sodium transport primarily through a cAMP‐dependent mechanism, enhancing the permeability of the apical membrane to sodium ions (Kortenoeven et al., 2015). In contrast, Gi‐mediated pathways inhibit this effect, demonstrating a regulatory balance between stimulatory and inhibitory signals in sodium transport. Notably, direct inhibition of cAMP production by Gi proteins can also influence other signaling pathways that affect sodium transport. The activation of proteinase‐activated receptor 2 (PAR2) stimulates Na^+^/K^+^‐ATPase activity and sodium reabsorption in the kidney epithelium, which operates through distinct signaling pathways that may interact with Gi signaling (Morla et al., 2008). Other Gi‐coupled receptors expressed in the collecting duct have been described to influence Na + excretion, such as the neuropeptide Y receptor Y1 (Npy1r) and the lysophosphatidic acid receptor 1 (Lpar1) (Chen et al., 2017; Poll et al., 2021; Tóth et al., 2024) however, further studies are needed to confirm the effect of Npy1r and Lpar1 activation in reducing ENaC activity. In addition, locally produced prostaglandin E2 (PGE2) secreted in the cortical collecting duct can mediate transepithelial Na^+^ transport, potentially affecting blood pressure (Mansley et al., 2020; Nasrallah et al., 2018).

Our group has previously investigated the role of Gq and Gs in regulating ENaC activity by utilizing the designer receptors exclusively activated by designer drugs (DREADD) technology, which showed to be an excellent tool to dissect the GPCR signaling regulating ENaC. Here, we investigated the role of Gi‐DREADD in regulating ENaC activity. Our results demonstrated that Gi‐DREADD expressed exclusively in renal PCs and activated by CNO reduced ENaC activity and significantly increased urinary Na^+^ excretion compared to CNO‐treated controls. These findings demonstrate for the first time that target activation of Gi signaling exclusively in PCs is sufficient to reduce ENaC activity and increase dependent urinary Na^+^ excretion in live animals.

In conclusion, the inhibition of adenylate cyclase by Gi subunits and the resultant decrease in cAMP levels demonstrated herein has significant clinical implications that need further clarification. Conditions such as heart failure, hypertension, and renal disorders may be influenced by alterations in Gi signaling pathways. Understanding these mechanisms can aid in the development of targeted therapies aimed at modulating renal function and in the control of hypertension.

## FUNDING INFORMATION

No funding information provided.

## ETHICS STATEMENT

All animal work were approved by the Institutional Animal Care and Use Committee of the University of Texas Health Science Center at San Antonio.
